# Juvenile Hormone III but Not 20-Hydroxyecdysone Regulates the Embryonic Diapause of *Aedes albopictus*

**DOI:** 10.3389/fphys.2019.01352

**Published:** 2019-10-25

**Authors:** Zachary A. Batz, Colin S. Brent, Molly R. Marias, Jennifer Sugijanto, Peter A. Armbruster

**Affiliations:** ^1^Department of Biology, Georgetown University, Washington, DC, United States; ^2^U.S. Arid Land Agricultural Research Center (USDA-ARS), Maricopa, AZ, United States

**Keywords:** embryonic diapause, hormonal regulation, juvenile hormone, ecdysone, *Aedes albopictus*, Diptera

## Abstract

Diapause is an alternative developmental trajectory allowing insects to enter dormancy and persist through predictable periods of seasonally unfavorable conditions. This crucial ecological adaptation defines the geographic and seasonal abundance of many insect pollinators, pests, and vectors. Understanding the hormonal changes by which insects coordinate the perception of external, diapause-inducing cues with the physiological mechanisms that lead to developmental arrest is a long-standing goal in biology. The hormonal regulation of diapause tends to vary by the life stage at which development arrest occurs; for example, diapause is typically regulated by ecdysteroids in larvae and pupae, and by juvenile hormones in adults. However, little is known about the hormonal control of embryonic diapause, particularly in Diptera. To address this fundamental gap, we directly measured 20-hydroxyecdysone (20HE) (via LC-MS/MS) and juvenile hormone III (JH3) (via GC-MS) in diapause and non-diapause eggs of the Asian tiger mosquito, *Aedes albopictus*. While 20HE abundance did not differ, diapause eggs had lower JH3 abundance than non-diapause eggs. These results are corroborated by transcriptional and manipulative evidence suggesting that reduced JH3 regulates diapause in this medically important mosquito.

## Introduction

The adaptive radiation of insects across diverse climates has been facilitated in part by the repeated evolution of diapause, an anticipatory developmental arrest in advance of unfavorable conditions (e.g., winter, dry season; Waterhouse and Norris, 1980; Tauber et al., 1986; Danks, 1987; Hodkova and Hodek, 2004). Diapause is typically initiated in response to a token environmental cue (e.g., seasonal change in photoperiod), maintained for a genetically determined period, and then terminated after favorable conditions have returned. Thus, diapause allows insects to align their growth and reproduction with local seasonal cycles (Tauber et al., 1986; Danks, 1987). Diapause is a key regulator of life history, geographic range, and seasonal abundance for many insect pollinators (e.g., Kemp and Bosch, 2005; Fründ et al., 2013), agricultural pests (e.g., Pullin et al., 1991; Li et al., 2012; Levine et al., 2015), and disease vectors (e.g., Denlinger and Armbruster, 2014; Gray et al., 2016). Additionally, diapause invokes widespread physiological modifications including accumulation of nutrient reserves, suppression of metabolic activity, increased anaerobic catabolism, cell cycle arrest, alterations in Wnt signaling, and upregulation of various stress tolerance mechanisms (Denlinger, 2002; Hahn and Denlinger, 2007, 2011; King and MacRae, 2015; Ragland and Keep, 2017). Understanding the regulatory mechanisms underlying diapause is a long-standing problem in organismal biology and will facilitate the development of new management approaches to key species.

Ecdysteroids and juvenile hormones regulate a wide range of physiological and developmental processes in insects (Gruntenko and Rauschenbach, 2008; Jindra et al., 2013; Palli, 2016), including diapause (Denlinger, 1985; Denlinger et al., 2012). The specific hormonal signal regulating diapause tends to vary by the stage at which developmental arrest occurs (Denlinger et al., 2012). For instance, developmental arrest during larval or pupal diapause is typically regulated by changes in ecdysteroid abundance (e.g., Ohtaki and Takahashi, 1972; Bowen et al., 1984; Richard et al., 1987). In contrast, developmental arrest during adult diapause is often regulated by changes in juvenile hormone abundance (e.g., Spielman, 1974; Schooneveld et al., 1977; Saunders et al., 1990 and see review in Denlinger et al., 2012).

A major gap exists in our understanding of the hormonal regulation of embryonic diapause, which is widely observed across Lepidoptera, Orthoptera, Hemiptera, and Diptera. Previous research has extensively documented endocrine regulation of embryonic diapause in the silkworm moth (*Bombyx mori*) by diapause hormone (Hasegawa, 1963; Yamashita and Hasegawa, 1966; Yamashita, 1996). However, this regulatory role for diapause hormone appears to be unique to *B. mori* and thus has provided little insight into the hormonal basis of embryonic diapause in other species (Denlinger et al., 2012). Beyond *B. mori*, the hormonal regulation of embryonic diapause has been established in just three species. The gypsy moth (*Lymantria dispar*) undergoes a developmental arrest initiated and maintained by higher abundance of ecdysteroids (Lee et al., 1997; Lee and Denlinger, 1997) while low ecdysteroid abundance regulates diapause in two locusts (*Chortoicetes terminifera*, *Locusta migratoria*; Gregg et al., 1987; Tawfik et al., 2002).

To date, no study has characterized hormone abundance during embryonic diapause of any Dipteran species. Here, we address this gap by investigating the hormonal regulation of embryonic diapause in the Asian tiger mosquito, *Aedes albopictus*. Over the past 30 years, this medically important vector (Paupy et al., 2009) has successfully invaded temperate regions worldwide (Benedict et al., 2007) due in part to an embryonic diapause that has facilitated survival during long-distance transport (Juliano and Lounibos, 2005; Diniz et al., 2017) and evolved rapidly to align the timing of developmental arrest with local climatic conditions (Urbanski et al., 2012). Temperate populations of *Ae. albopictus* enter a maternally regulated, photoperiodic diapause. Under short, autumnal daylengths, females produce eggs which complete embryonic development but remain refractory to hatching stimuli as pharate larvae inside the chorion of the egg until the following spring. Over the past decade, the transcriptional regulation of embryonic diapause in *Ae. albopictus* has been extensively characterized (Urbanski et al., 2010; Poelchau et al., 2011, 2013a,b; Huang X. et al., 2015; Batz et al., 2017) but the hormonal regulation of this crucial adaptation remains unknown. We quantified 20-hydroxyecdysone (20HE) and juvenile hormone III (JH3) in diapausing and non-diapausing eggs at three time points that bracket key stages of embryonic development and interpreted our results in the context of previously collected RNAseq (Poelchau et al., 2013a,b) and manipulative data (Suman et al., 2015).

## Materials and Methods

### Egg Collection

All measurements used F_11_ eggs from a laboratory colony of *Ae. albopictus* established in August 2015 from over 200 larvae collected in Manassas, VA, United States. Prior to this experiment, the population was maintained under previously described conditions [Armbruster and Conn, 2006; long-day photoperiod (LD) 16:8 L:D, 21°C, 80% RH]. To generate egg samples for this experiment, F_10_ larvae were maintained in 5.5 L Sterlite containers at a density of approximately 250 larvae per 2.5 L deionized (DI) water with 5 mL of food slurry (Armbruster and Conn, 2006) under LD photoperiod at 21°C and 80% RH. Every Monday–Wednesday–Friday, larvae were filtered through a fine mesh net then transferred to a clean 5.5 L Sterlite container; pupae were distributed to adult cages maintained under either diapause-averting LD photoperiod or diapause-inducing, short-day photoperiod (SD, 8:16 L:D).

Adult females were provided weekly bloodmeals and an oviposition cup lined with unbleached paper towel and half-filled with DI water until oviposition ceased. Eggs were collected daily, maintained on a wet paper towel for 48 h, then air dried and stored in containers under SD conditions. At 5, 7, and 11 days post-oviposition (dpov), collected eggs were weighed to the nearest 1 μg (mean: 11.460 mg, range: 9.1–19.5 mg) on a MX5 microbalance (Mettler-Toledo, Columbia, OH, United States), placed in 1.5 mL tubes, snap-frozen in liquid nitrogen between 11 am and 12 pm (Zeitgeber time 3–4 h), and stored at −80°C. A subset of eggs was retained to confirm that LD and SD photoperiod conditions stimulated the production of non-diapause and diapause eggs, respectively (see Urbanski et al., 2012).

We sampled eggs at 5, 7, and 11 dpov for two reasons. First, *Ae. albopictus* initiates diapause (*sensu* Koštál, 2006; Koštál et al., 2017) following completion of embryonic development (Mori et al., 1981). Under the conditions utilized in our experiments, *Ae. albopictus* embryos undergo segmentation at approximately at 5 dpov and non-diapause embryos are competent to hatch at approximately 7 dpov (Poelchau et al., 2013a). By 11 dpov, developmental arrest is firmly established in diapause embryos (Poelchau et al., 2013b). Thus, we chose to compare diapause and non-diapause embryos at time points bracketing the period of hatching competency, including the establishment of developmental arrest during diapause. Second, we chose time points that were similar to those used for previous RNAseq experiments of embryos (Poelchau et al., 2013a, b) so that these data could be leveraged to interpret our current results. These RNAseq results were previously found to be well-correlated with gene expression results obtained via qRT-PCR (Poelchau et al., 2013a).

### Ecdysteroid Quantification

To extract ecdysteroids, egg samples were placed in a borosilicate glass 0.5 mL conical bottomed micro tissue grinder tube (Wheaton, Millville, NJ, United States) with 1 μg of farnesol (Sigma-Aldrich, St. Louis, MO, United States) added as an internal standard. Eggs were manually homogenized three times with a Teflon pestle in 300 μL HPLC-grade chilled MeOH then spun at 12,000 RCF for 10 min in a refrigerated (4°C) centrifuge. The resulting supernatant was pooled then eluted through aluminum oxide columns twice with chilled 90% MeOH and once with MeOH. Samples were dried down in a refrigerated (4°C) vacuum centrifuge, resuspended in 20 μL MeOH, and stored at −80°C until analysis. Positive controls were prepared by adding 1 μg each of 20HE (Sigma-Aldrich) and farnesol to empty tubes that were otherwise processed identically. Sample analysis of ecdysteroid concentration was performed using a Micromass Quattro Micro LC-MS/MS (Waters Co., Milford, MA, United States). Injection volumes were 5.0 μL, with separations performed using ACQUITY UPLC BEH C18 2.1 mm by 50 mm columns with a 1.7 μm stationary phase (Waters Co.). Operating conditions for LC runs were a mobile phase flow rate of 0.37 mL min^–1^ with a binary mobile phase of 0.1% formic acid in acetonitrile and 0.1% formic acid in water. Initial conditions were 1:99 acetonitrile:water, followed by isocratic flow for 0.3 min. At 0.3 min, a linear gradient from 1:99 to 99:1 acetonitrile:water was applied over 4.2 min, followed by 1.0 min isocratic flow at 99:1 acetonitrile:water, after which the mobile phase returned to 1:99 acetonitrile:water. Mass spectrometer settings were electrospray positive, with a desolvation temperature of 350°C. 20HE and farnesol eluted at 2.89 and 4.97 min, respectively, and were quantified using multiple reaction monitoring of characteristic transitions: 481.22 (*m/z*) > 445.24 (*m/z*), and 205.41 (*m/z*) > 121.09 (*m/z*), respectively. The detection limit of the assay is approximately 5 pg.

### Juvenile Hormone Quantification

Each egg sample was placed in a borosilicate glass 0.5 mL conical-bottomed micro tissue grinder tube (Wheaton) with 1 μg of farnesol (Sigma-Aldrich) added as an internal standard. Eggs were manually homogenized three times with a Teflon pestle in 500 μL HPLC grade chilled hexane, then spun at 12,000 RCF for 10 min in a refrigerated (4°C) centrifuge. The hexane fractions were recombined in a clean borosilicate glass vial and dried by vacuum centrifugation. JH was quantified using gas chromatography/mass spectrometry as previously described (Brent and Vargo, 2003). Briefly, the residue was washed out of the vials with three rinses of hexane and added to borosilicate glass columns filled with aluminum oxide. To filter out contaminants, samples were eluted through the columns successively with hexane, 10% ethyl ether-hexane and 30% ethyl ether-hexane. After drying, samples were derivatized by heating at 60°C for 20 min in a solution of methyl-d alcohol (Sigma-Aldrich) and trifluoroacetic acid (Sigma-Aldrich). Samples were dried down, resuspended in hexane, and again eluted through aluminum oxide columns. Non-derivatized components were removed with 30% ethyl ether. The JH derivative was collected into new vials by addition of 50% ethyl-acetate–hexane. After drying, samples were resuspended in hexane, then analyzed using an HP 7890A Series GC (Agilent Technologies, Santa Clara, CA, United States) equipped with a 30 m × 0.25 mm Zebron ZB-WAX column (Phenomenex, Torrance, CA, United States) coupled to an HP 5975C inert mass selective detector. Helium was used as a carrier gas. JH form was confirmed by first running test samples in SCAN mode for known signatures of JH 0, JH I, JH II, JH3, and JH3 ethyl; JH3 was confirmed as the primary endogenous form in this species. Subsequent samples were analyzed using the MS SIM mode, monitoring at *m/z* 76 and 225 to ensure specificity for the d3-methoxyhydrin derivative of JH3. Total abundance was quantified against a standard curve of derivatized JH3 and adjusted for the starting mass of eggs. The detection limit of the assay is approximately 1 pg.

### Statistical Analyses

Analyses were performed in R (R Core Team, 2019). JH3 abundance was log-transformed and analyzed using ANOVA to test for effects of diapause status, age, and their interaction. 20HE abundance was non-linear and fit using two polynomial regression models: a full model including age and developmental status (diapause, non-diapause), and a reduced model containing only age. Models were compared using ANOVA to determine if including developmental status significantly improved explanatory power.

Transcriptional analyses utilized previously collected RNAseq data from diapause and non-diapause *Ae. albopictus* eggs (Supplementary Dataset 1; Poelchau et al., 2013a, b). Differentially expressed genes at 3, 6, and 11 dpov were investigated by gene set enrichment analyses (Mootha et al., 2003) using the Piano package (Väremo et al., 2013) set to default parameters followed by FDR correction. A reference gene set was annotated with the biological process GO terms assigned on VectorBase (Giraldo-Calderón et al., 2015). JH3 and ecdysteroid synthesis pathway genes as well as JH3 receptors and degradation genes were manually annotated according to previous literature (Bai et al., 2007; Li et al., 2011; Nouzova et al., 2011; Niwa and Niwa, 2014; Matthews et al., 2016). Additionally, we annotated JH3 and ecdysteroid-inducble genes based on gene descriptions provided in VectorBase (Giraldo-Calderón et al., 2015). A complete overview of these gene sets is provided in Supplementary Dataset 1.

## Results

**FIGURE 1:**
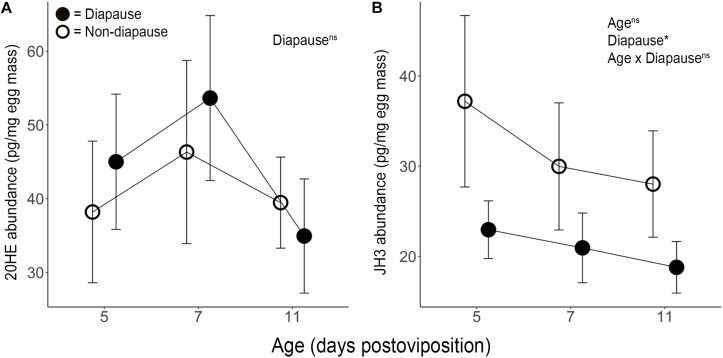
Mean ± SE **(A)** 20-hydroxyecdysone (*n* = 5–10) and **(B)** juvenile hormone III (*n* = 11–12) abundance per mg of diapause (●) and non-diapause (○) eggs. The results of ANOVA shown in the upper right indicate effects of age, diapause status and their interaction. ^∗^Indicates *p* < 0.05 and “ns” indicates *p* > 0.05.

Mean diapause incidence was 95.9% (range: 90.5–100%) under SD photoperiod and 5.4% (range: 1.8–9.5%) under LD photoperiod (Supplementary Dataset 2); thus, our photoperiodic treatments induced the expected diapause responses. 20HE peaked under both diapause and non-diapause conditions at 7 dpov but including diapause status as a factor in a quadratic model for 20HE abundance did not improve the explanatory power of the model (Figure 1A and Supplementary Dataset 3; *F*_3,43_ = 0.21, *p* = 0.890). These results indicate that diapause status does not significantly explain variation in 20HE titer. Pathway analysis of RNAseq indicated that neither the 20HE synthesis pathway nor the 20HE-inducible genes were significantly altered under diapause vs. non-diapause conditions at 3, 6 or 11 dpov (Table 1).

**TABLE 1:** Gene set enrichment analysis results, see Supplementary Dataset 1 for a complete summary of these gene sets.

		**Early preparation (3 dpov)**			**Late preparation (6 dpov)**			**Early diapause (11 dpov)**		
				
**Group**	**Genes (n)**	**# Up**	**# Down**	***p*-value^∗^**	**# Up**	**# Down**	***p*-value**	**# Up**	**# Down**	***p*-value**
20HE synthesis	8	5	3	1.000	1	7	0.875	6	1	1.000
20HE inducible	27	13	14	0.161	12	15	1.000	11	13	0.184
JH synthesis	13	1	10	0.033	3	8	0.996	6	6	0.943
JH inducible	15	7	8	0.963	2	13	0.018	7	8	0.928

*^∗^*p-*value estimates likelihood that cumulative change in expression across a gene set are significantly correlated with diapause (see Subramanian et al., 2005).*

**FIGURE 2:**
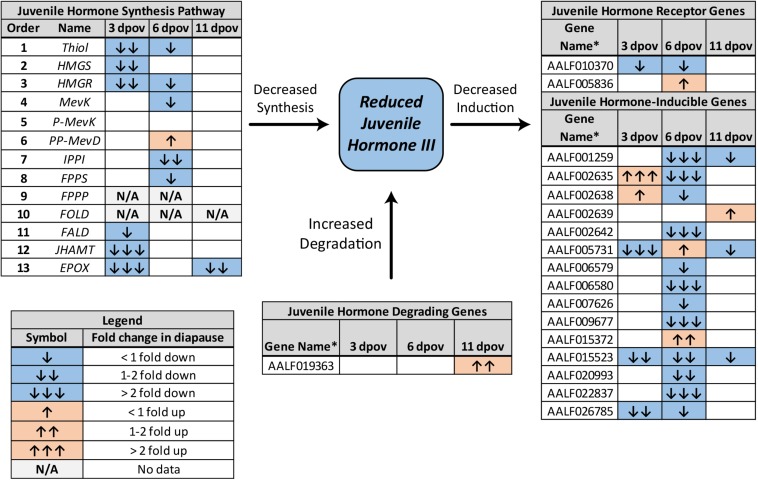
Summary of differential expression of genes involved in JH3 synthesis, degradation, and induction for *Ae. albopictus* (Poelchau et al., 2013a, b). Significant diapause-induced reductions (blue) or increases (orange) are indicated by shaded cells. The differential magnitude is indicated by arrows as described in the legend in the lower left. ^∗^Genes without common names are identified by VectorBase ID number.

JH3 was confirmed to be the primary form of juvenile hormone in this species. JH3 abundance was significantly reduced in diapause embryos (Figure 1B and Supplementary Dataset 3; *F*_1,64_ = 5.08, *p* = 0.028). However, age (*F*_2,64_ = 0.66, *p* = 0.518) and the interaction of diapause status and age (*F*_2,64_ = 0.13, *p* = 0.883) did not affect JH3 levels. Pathway analysis of RNAseq data indicated that the JH3 synthesis pathway was significantly repressed at 3 dpov and JH-inducible genes were significantly repressed at 6 dpov (Figure 2 and Table 1).

## Discussion

Diapause is a widespread adaptation that allows insects to align their growth and reproduction with seasonally favorable conditions (Tauber et al., 1986; Danks, 1987). Understanding the hormonal changes by which insects coordinate the perception of external, diapause-inducing cues with the physiological mechanisms that lead to developmental arrest is a long-standing goal in biology (Lees, 1956; Denlinger, 2002). Decades of research have led to the identification of common hormonal strategies for diapause at the larval, pupal, and adult stages; however, the hormonal regulation of embryonic diapause remains largely unknown, particularly in Diptera (Denlinger, 1985, 2002; Denlinger et al., 2012). In this study, our direct measurement of reduced JH3 abundance in diapause is corroborated by previous gene expression profiling (Poelchau et al., 2013a, b) and manipulative experiments (Suman et al., 2015) supporting the hypothesis that JH3 regulates embryonic diapause in this species.

### Ecdysteroids

In the present study, we directly measured ecdysteroid abundance at three time points under diapause and non-diapause conditions in embryos of the mosquito *Ae. albopictus.* Consistent with previous results from *Toxorhynchites amboinensis* (Russo and Westbrook, 1986), 20HE levels peaked near the conclusion of embryonic development (7 dpov; Figure 1A). However, 20HE titer did not significantly differ between diapause and non-diapause eggs. Furthermore, neither the 20HE synthesis pathway nor 20HE-inducible genes underwent any significant, coordinated changes in gene expression (Table 1). We conclude that, in contrast to previously examined embryonic diapauses in Lepidoptera and Orthoptera (Hasegawa, 1963; Yamashita and Hasegawa, 1966; Gregg et al., 1987; Yamashita, 1996; Lee and Denlinger, 1997; Lee et al., 1997; Tawfik et al., 2002), 20HE does not regulate diapause initiation in *Ae. albopictus*.

### Juvenile Hormone

In contrast, we observed approximately two-fold lower JH3 abundance in diapause embryos (Figure 1B). While smaller in magnitude than differences in JH3 abundance detected under diapause vs. non-diapause conditions in insects that diapause at other life stages (e.g., Yin and Chippendale, 1979; Walker and Denlinger, 1980; Readio et al., 1999), our direct measurements are corroborated by transcriptional evidence (Figure 2). For example, the JH3 synthesis pathway is significantly repressed during early diapause preparation (3 dpov). Supporting our interpretation of these results, previous studies have found that reduced expression of genes along the JH3 synthesis pathway is strongly correlated with lower JH3 abundance in both *Aedes aegypti* (Nouzova et al., 2011; Rivera-Perez et al., 2014) and *Diploptera punctata* (Huang J. et al., 2015). JH-inducible genes are also significantly repressed during late diapause preparation (6 dpov; Table 1), suggesting that diapause-destined embryos have a limited capacity to respond to JH3 signaling near the time that non-diapause embryos become competent to hatch. Furthermore, gene expression for a JH3-degrading enzyme (JH esterase) is significantly upregulated in early diapause maintenance (11 dpov; Figure 2) as well as later in diapause maintenance (21 and 40 dpov; Poelchau et al., 2013b) suggesting JH3 may remain at low abundance throughout diapause in this species.

Reduced JH3 abundance during developmental arrest is somewhat counterintuitive because low juvenile hormone titer is typically associated with progressive molts, particularly from the larval to pupal stage (Palli, 2016). However, application of a JH3 analog (pyriproxyfen) to diapause *Ae. albopictus* eggs accelerates the rate of diapause termination in a dose-dependent manner (Suman et al., 2015). At the most effective dosage, approximately 80% of pyriproxyfen exposed eggs terminate diapause by 30 dpov compared to just 1% diapause termination by 80 dpov for control eggs (Suman et al., 2015). Alterations in JH3 abundance can also generate an embryonic diapause-like phenotype in other species. For example, in *B. mori*, experimental reduction of JH3 synthesis by *JHAMT* knockout results in fully developed pharate larvae that are unresponsive to hatching stimuli; this phenotype can be rescued in a dose-dependent manner by application of either extracted JH3 or methoprene, a JH3 analog (Nakao et al., 2015). Similarly, experimental supplementation of another juvenile hormone analog (RO-20-3600) to newly deposited *Drosophila melanogaster* eggs produces pharate larvae which are mobile within the egg but fail to hatch (Smith and Arking, 1975). Together, these data indicate that juvenile hormone abundance likely contributes to the regulation of hatching responses in diverse insects. Embryonic diapause at the pharate larval stage is defined by a failure to respond to hatching stimuli thus, changes in of juvenile hormone abundance may represent a common hormonal strategy for regulating this form of diapause.

In concert with previous transcriptional and manipulative data, our results strongly implicate reduced JH3 abundance as the likely regulator of embryonic diapause in *Ae. albopictus*. This research is the first direct quantification of hormone abundance during embryonic diapause in any Dipteran species and represents an important step toward clarifying the hormonal regulation of this crucial adaptation to adverse environments. Characterizing the linkage between external stimuli and developmental arrest via hormonal signaling remains a major challenge (Denlinger, 1985; Denlinger et al., 2012). Elucidating these pathways in a variety of species will provide critical insight for fundamental and applied problems including understanding the molecular and physiological basis of life history evolution, predicting species responses to climate change, and identifying novel targets for management of pest and vector species (Denlinger, 2008).

## Data Availability Statement

All datasets generated for this study are included in the article/Supplementary Material.

## Author Contributions

ZB, CB, and PA contributed to the conception, design of the study, and prepared the initial draft of the manuscript. ZB, MM, and JS performed the animal husbandry and collected the egg samples. CB performed the LC-MS/MS and GC-MS analyses. ZB performed the statistical analyses. All authors contributed to the manuscript revision and approved the submitted version.

## Conflict of Interest

The authors declare that the research was conducted in the absence of any commercial or financial relationships that could be construed as a potential conflict of interest.
